# Observational multi-centre, prospective study to characterize novel pathogen-and host-related factors in hospitalized patients with lower respiratory tract infections and/or sepsis - the “TAILORED-Treatment” study

**DOI:** 10.1186/s12879-018-3300-9

**Published:** 2018-08-07

**Authors:** C. B. van Houten, K. Oved, E. Eden, A. Cohen, D. Engelhard, S. Boers, R. Kraaij, R. Karlsson, D. Fernandez, E. Gonzalez, Y. Li, A. Stubbs, E. R. B. Moore, J. P. Hays, L. J. Bont

**Affiliations:** 1 Division of Paediatric Immunology and Infectious Diseases, University Medical Centre Utrecht, Utrecht University, Office KC.03.063.0, P.O. Box 85090, 3508 AB Utrecht, The Netherlands; 2 MeMed, Tirat Carmel, Israel; 3 Division of Paediatric Infectious Disease Unit, Hadassah-Hebrew University Medical Centre, Jerusalem, Israel; 4 Department of Medical Microbiology and Infectious Diseases, Erasmus University Medical Centre (Erasmus MC), Rotterdam, the Netherlands; 5 Department of Internal Medicine, Erasmus University Medical Centre (Erasmus MC), Rotterdam, the Netherlands; 6 Department of Infectious Diseases, Institute of Biomedicine, Sahlgrenska Academy, University of Gothenburg, Gothenburg, Sweden; 7 Noray Bioinformatics, Derio, Spain; 8 Department of Pathology, Clinical Bioinformatics Unit, Erasmus University Medical Centre (Erasmus MC), Rotterdam, the Netherlands; 9 University of Gothenburg, Gothenburg, Sweden

**Keywords:** Antibiotic resistance, Sepsis, Rapid diagnostics, Lower respiratory tract infections

## Abstract

**Background:**

The emergence and spread of antibiotic resistant micro-organisms is a global concern, which is largely attributable to inaccurate prescribing of antibiotics to patients presenting with non-bacterial infections. The use of ‘omics’ technologies for discovery of novel infection related biomarkers combined with novel treatment algorithms offers possibilities for rapidly distinguishing between bacterial and viral infections. This distinction can be particularly important for patients suffering from lower respiratory tract infections (LRTI) and/or sepsis as they represent a significant burden to healthcare systems. Here we present the study details of the TAILORED-Treatment study, an observational, prospective, multi-centre study aiming to generate a multi-parametric model, combining host and pathogen data, for distinguishing between bacterial and viral aetiologies in children and adults with LRTI and/or sepsis.

**Methods:**

A total number of 1200 paediatric and adult patients aged 1 month and older with LRTI and/or sepsis or a non-infectious disease are recruited from Emergency Departments and hospital wards of seven Dutch and Israeli medical centres. A panel of three experienced physicians adjudicate a reference standard diagnosis for all patients (i.e., bacterial or viral infection) using all available clinical and laboratory information, including a 28-day follow-up assessment. Nasal swabs and blood samples are collected for multi-omics investigations including host RNA and protein biomarkers, nasal microbiota profiling, host genomic profiling and bacterial proteomics. Simplified data is entered into a custom-built database in order to develop a multi-parametric model and diagnostic tools for differentiating between bacterial and viral infections. The predictions from the model will be compared with the consensus diagnosis in order to determine its accuracy.

**Discussion:**

The TAILORED-Treatment study will provide new insights into the interplay between the host and micro-organisms. New host- or pathogen-related biomarkers will be used to generate a multi-parametric model for distinguishing between bacterial and viral infections. This model will be helpful to better guide antimicrobial therapy for patients with LRTI and sepsis. This study has the potential to improve patient care, reduce unnecessary antibiotic prescribing and will contribute positively to institutional, national and international healthcare economics.

**Trial Registration:**

NCT02025699. Registration Date: January, 1, 2014.

**Electronic supplementary material:**

The online version of this article (10.1186/s12879-018-3300-9) contains supplementary material, which is available to authorized users.

## Background

### The burden of antibiotic misuse

Antibiotics are the most prescribed class of drugs worldwide [1]. However, 30–50% of antibiotics are estimated to be prescribed inappropriately, making antibiotics also the most misused drug class [1–5]. Antibiotic overuse, i.e. prescribing antibiotics to treat a non-bacterial disease, is a serious problem. For example, in the US over 80 million antibiotic prescriptions are given annually for viral infections in the outpatient setting [6]. This may cause the emergence of adverse events (AEs) such as allergic reactions and antibiotic-associated diarrhoea [7]. Importantly, antibiotic overuse has been linked to the emergence of resistant strains of bacteria, by the Center for Disease Control considered as “one of the world’s most pressing health problems in the 21^st^ century” [8, 9]. Annually, 23,000 deaths due to resistant bacteria are expected in the US alone [8, 10]. Resistant bacteria are projected to cause over 10 M annual deaths worldwide by 2050, surpassing cancer as the leading cause of death [8, 11]. A second type of antibiotic misuse is underuse, i.e. delayed or no antibiotic treatment in case of a bacterial disease, from which a patient could have benefited [12–17]. Although reducing the risk of antibiotic-related AEs [18, 19], underuse of antibiotics may lead to a prolonged disease duration and an increased rate of complications that could have been avoided with early antimicrobial treatment [19–21]. For example, up to 15–40% of adult patients hospitalized for bacterial pneumonia in the US receive delayed or no antibiotic treatment [14, 15]. Finally, applying the wrong antibiotic spectrum to treat a bacterial disease is the third type of antibiotic misuse. For example, administering third generation cephalosporins instead of macrolides to treat a respiratory infection caused by atypical bacteria.

### Health economics consequences

Antibiotic overuse has also several health economics consequences. For instance, the annual cost of unnecessary antibiotic prescriptions for adult respiratory infections in the US is estimated to be $1.1 billion [22]. In addition, there are also indirect costs, including the costs of treating preventable antibiotic-related AEs and the costs of prolonged hospital stay as a result of AEs. Lastly, are the costs related to the emergence of antibiotic-resistant bacterial strains. The cost of treating patients with antibiotic-resistant bacteria is estimated at $16–26 billion annually in the US [23–25] and over €1.5 billion in the EU [26]. The health economics consequences of underuse of antibiotics include the costs of treating preventable complications and a prolonged disease duration resulting from a delayed or no antibiotic treatment [19–21].

### Antibiotics in respiratory tract infections and sepsis

Respiratory tract infections are one of the most common causes of hospital and outpatient visits in the EU, comprising 1 out of 3 admissions annually [9]. In patients with mild respiratory tract infections, over prescription of antibiotics in general and prescription of broad spectrum antibiotics are large problems. Broad spectrum antibiotics account for approximately half of all antibiotic prescribing for children with acute RTI [27], with the above mentioned consequences [8]. Sepsis-related hospital admissions are less frequent but represent a significant burden to healthcare systems in terms of adverse patient outcomes and the need for rapid, but effective, intervention strategies [28]. In septic patients the key clinical challenge is to ensure that the correct antimicrobial treatment is administered, and that this therapy is administered as soon as possible [29].

### Limitations of current diagnostic tools

**Fig. 1:**
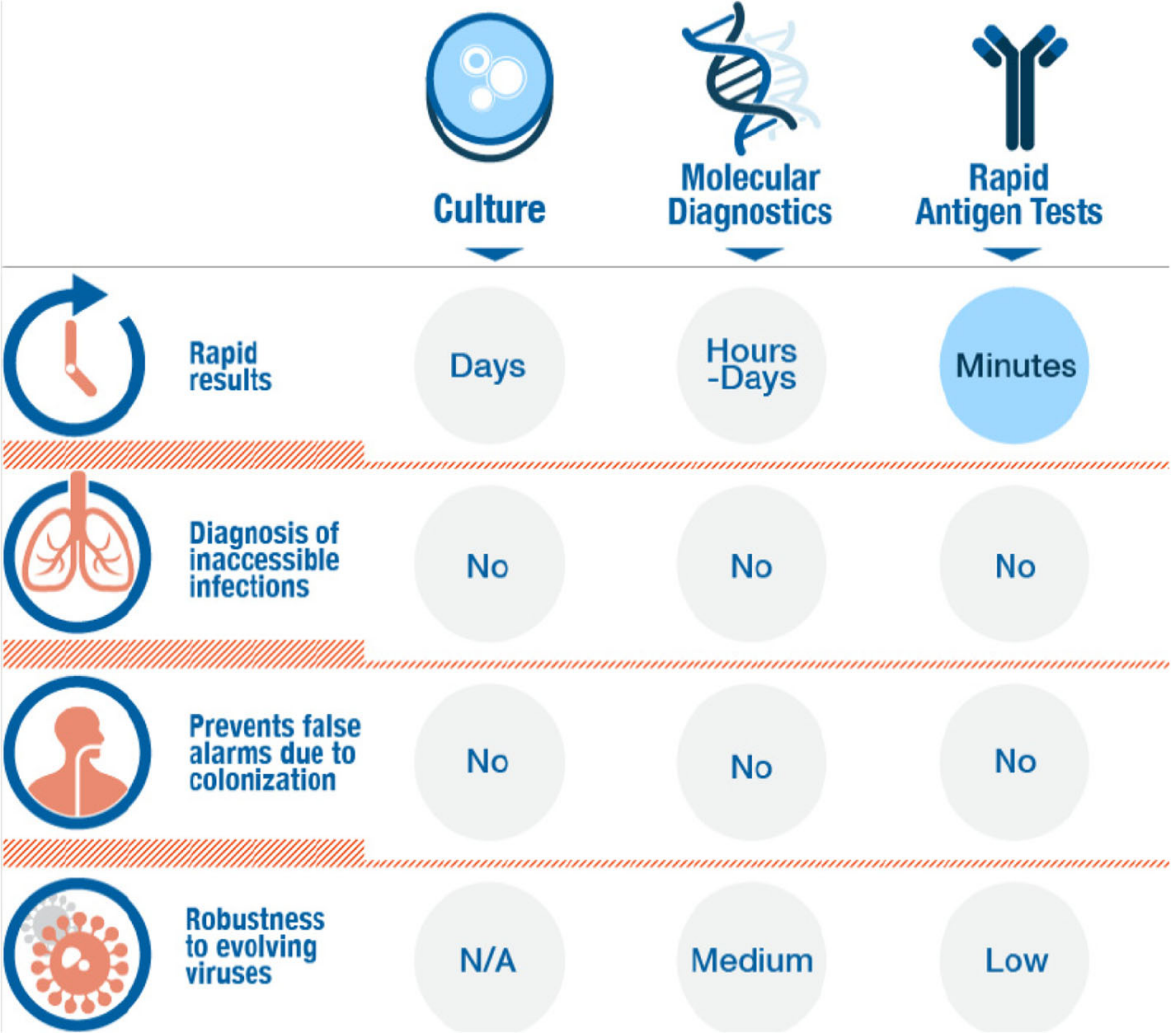
Limitations of current diagnostic tools

Although the current diagnostic tools for facilitating appropriate use of antibiotics (such as culture-, PCR-, and immunoassay-based) may be valuable in some clinical situations, they have major limitations (Fig. 1). First, existing diagnostic tools often require hours to days to provide information, whereas physicians need to decide whether to prescribe antibiotics within shorter time periods. Second, available diagnostic technologies usually require direct sampling of the pathogen. Such sampling is often not feasible if the infection site is inaccessible (e.g., pneumonia, sinusitis, middle-ear infection, etc.). A third limitation is that available technologies usually search for the presence of specific bacteria or viruses. However, many types of bacteria and viruses could be present as part of the natural flora without causing an infection. For example, the important respiratory tract pathogenic bacteria *S. pneumoniae* is also part of the upper respiratory tract natural flora in up to 68% and 15% of healthy children and adults, respectively [30] and respiratory viruses were detected in 35.4% of a-symptomatic children [31]. Finally, diagnostic solutions that were developed for detection of a specific pathogenic strain, often fail to detect the constantly evolving and emerging strains of the same family of pathogens, for example rapid Influenza tests showed low sensitivity, 45%, during the 2009 H1N1 influenza A viral outbreak [32]. These limitations lead to a ‘diagnostic gap’, which in turn often leads physicians to either over-prescribe (e.g., ‘just-in-case’ approach) or under-prescribe (e.g., ‘watchful waiting’ approach) antibiotics [18–20], both of which adversely influence patient care and health economics.

### The TAILORED-treatment project

The health and health economics consequences of antibiotic misuse highlight the need for a diagnostic tool that would help physicians to use antibiotics appropriately. Ideally, this diagnostic tool should provide a diagnosis that can accurately and rapidly differentiate between bacterial and viral infections. However, as previously described, current diagnostic tools are often inadequate (Fig. 1). Therefore, the TAILORED-Treatment consortium was established to develop new tools aimed at increasing the effectiveness of antibiotic therapy, reduce adverse events, and limit the emergence of antimicrobial resistance in children and adults. In reality, targeted antimicrobial therapy can most effectively be achieved by utilizing personalized data to facilitate a tailored and optimized approach to individual patient treatment. This can best be achieved by utilizing knowledge gained from both host-centred and pathogen-centred parameters during health and disease. Unfortunately, these parameters have traditionally, tended to be measured independently, and used on an ad hoc basis without careful integration for the best treatment of the patient. However, recent advances in the development of high-throughput and sensitive molecular-based technologies, improved databases, and bioinformatics analysis tools, rapidly enabling that the goal of personalized medicine and treatment can be reached. Unfortunately, however, there currently exists a ‘technological gap’ between recent state-of-the-art methodologies (for example with respect to gaining new insights into novel host-pathogen interactions) and laboratory-to-bedside results to benefit patients, physicians and society as a whole [33]. The observational TAILORED-Treatment project is designed to bridge this technological gap in order to generate novel insights and innovations that are readily exploitable in the field of personalized medicine and infectious diseases.

### Study objectives

#### Primary objective

To develop a novel multi-parametric diagnostic model for the management of patients with LRTI and/or sepsis that is based on novel pathogen- and host-related factors.

#### Secondary objectives

1. Identify individual as well as sets of blood host biomarkers for differentiating between bacterial, and viral aetiologies and determine the sensitivity and specificity of these biomarkers in distinguishing between the different infection types;
2. Identify individual as well as sets of blood host biomarkers for differentiating between bacterial subtypes, namely Gram positive, Gram negative and atypical bacteria;
3. Characterize the temporal dynamics of the host-pathogen interactions based on multiple sampling before and during antimicrobial treatment;
4. Characterize the respiratory microbiome in patients presenting with LRTI and/or sepsis;
5. Develop a mass spectrometry based, rapid detection technique for identification of microbial pathogens and antimicrobial resistances in clinical samples;
6. Develop a personalized treatment algorithm for tailored antimicrobial therapy that integrates clinical, molecular and biochemical data;
7. Define genetic mechanisms underlying the differential host response between patients with viral versus bacterial infection.

## Methods

### General study design

**Fig. 2:**
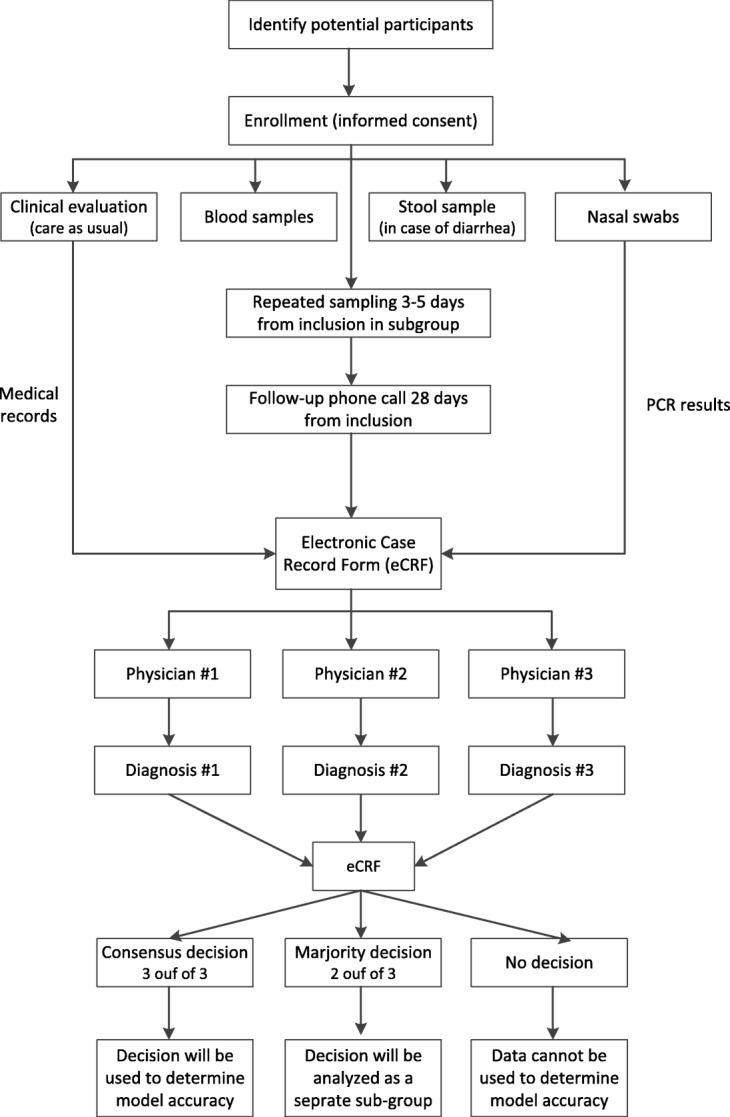
Schematic diagram of the study workflow

In this prospective, multi-centre, observational study, an expected total number of 1200 paediatric and adult patients aged 1 month and older with suspected LRTI and/or sepsis or a non-infectious disease are recruited, in order to develop a diagnostic model to differentiate between bacterial and viral infections. The study will generate a model for patients with LRTI and a separate model for patients with sepsis. The prediction of the model obtained from each patient will be compared with a consensus diagnosis by a panel of three experts in order to determine its accuracy (Fig. 2). The study is a non-interventional study, and therefore, the diagnostic prediction of the model will not be used for any clinical or diagnostics decision making. Local data monitor plans were used and executed by local, independent monitors.

### Study participants

Patients aged 1 month and older that attend the Emergency Department (ED) and hospital wards of seven Dutch and Israel medical centres (secondary and tertiary) due to a suspected LRTI and/or sepsis that agree (or their legal guardians agrees) to sign an informed consent and meet the inclusion criteria and none of the exclusion criteria (Table 1), are eligible to participate in the study. Informed consent is asked by the study team (i.e. research nurses or medical doctor). Sepsis criteria are defined according to published criteria [34]. Patients receive care as usual. The non-infectious disease group include patients with clinical signs of a non-infectious disease.

**Table 1:** Eligible criteria for Tailored Treatment study

Inclusion criteria	Exclusion criteria
Age ≥ 1 month	Proven or suspected HIV, HBV, or HCV infection
Symptoms ≤8 days	Post-transplant
*LRTI; ≥ 2 signs*	Congenital immune deficiency
Respiratory rate > 20/min^a^	Obvious alternative causes of respiratory distress
Chest cough	Nosocomial LRTI (> 3 days after hospitalization)
Nasal flaring	Other febrile infection during the past 3 weeks
Retractions	Active (haematological) malignancy
Rales	Immune-suppressive or immune-modulating therapies
Expiratory wheeze	Moderate to severe psychomotor retardation
Decreased breath sounds	Moderate to severe congenital metabolic disorder
*Sepsis* ^b^ *; ≥ 2 signs* ^c^	
Heartrate > 90/min	
Respiratory rate > 20/min	
Core body temperature > 38 °C or < 36 °C	
White blood cell count > 12,000 cells/ μl or < 4000/ μl	

^a^WHO age-specific criteria for tachypnea will be used for children [41]

^b^Age-specific criteria for sepsis criteria according to the guidelines of the International Paediatric Sepsis Consensus Conference 2005 will be used for children [42]

^c^One of which must be abnormal temperature or white blood cell count

*HIV* human immunodeficiency virus, *HBV* hepatitis B virus, *HCV* hepatitis C virus

### Data/sample collection

#### Clinical data

Clinical evaluation and management is performed according to standard institutional procedures (Fig. 2). Collected clinical data, includes demographics, medical history, clinical symptoms, physical examination, disease course, laboratory measurements, as well as more advanced diagnostic information including microbiological (including an additional multiplex PCR), and serological investigations. A follow-up phone call 28-day after recruitment, defines the end of follow-up and generates data to be used by the expert panel (e.g. maximum disease severity, total use of antibiotics, Additional file 1).

#### Sample collection

For study purposes two blood samples, two nasal swabs and (in case of diarrhoea) a stool sample are collected (Fig. 2). Blood and nasal swabs sampling at two sampling points (at inclusion and after 3–5 days) is performed in hospitalized Dutch children in order to monitor the temporal dynamics of the host-pathogen interactions. One nasal swab sample (Universal Transport Medium, Copan, USA) is used for PCR based microbiological investigations performed for the establishment of patient diagnosis (only at inclusion). The PCR results are included in the eCRF and are available to the TAILORED-Treatment expert panel while determining the diagnosis of the patient (Fig. 2). The second nasal swab is collected in Transport Medium suitable for bacterial culturing (e-swab, Liquid Amies Medium, Copan), this sample is used for respiratory microbiome investigations and to develop a rapid detection technique for identification of microbial pathogens and antimicrobial resistances, based on mass spectrometry and proteomics for discovery of peptide biomarkers. Serum samples are used for different proteomic measurements, leukocytes are isolated for discovery of RNA-based biomarkers. Following RNA isolation, remaining DNA is used for GWAS studies. GWAS analyses is performed in the Netherlands, as Institutional review board (IRB) approval for genetic testing is not obtained in Israel. DNA collection is mentioned explicitly in the patient information. Blood samples are collected from children with a non-infectious disease only if blood sampling is required as part of their routine care. In cases of diarrhoea on enrolment, stool samples are collected. These samples will be evaluated to identify *Clostridium difficile* infections.

#### ‘Proteotyping’

Shotgun proteomic detection and identification of microorganisms, or ‘proteotyping’, relies on recognition of species-unique peptides by tandem mass spectrometry (MS), allowing discrimination of species facilitated at amino acid level resolution. The recent evolution of mass spectrometers, having high sensitivity, accuracy and resolution, enables detection of almost the complete proteome of a microorganism. With such analytical means, it is feasible to determine directly, within a clinical sample, the species identity, the sub-species strain type and the factors expressing virulence and antimicrobial resistance [35]. Clinical samples are processed by first removing human biomass and subsequently collecting bacteria. The proteins of the collected bacteria were processed by the LPI™ FlowCell or in-solution digestion protocols followed by liquid chromatography tandem MS (LC-MS/MS) analysis. The recently developed technology called Lipid-based Protein Immobilization (LPI™) is based on immobilization of biological material within a flow cell, followed by digestion of exposed proteins by an enzyme, such as trypsin [36–38]. The MS-based proteomics methodology has been applied successfully, without prior cultivations to the analyses of clinical nasal swab samples from the Sahlgrenska University Hospital that have been confirmed positive for a respiratory infectious bacterial species, i.e., *S. pneumoniae, H. influenza, M. catarrhalis and S. aureus* (unpublished data).

#### Central database

**Fig. 3:**
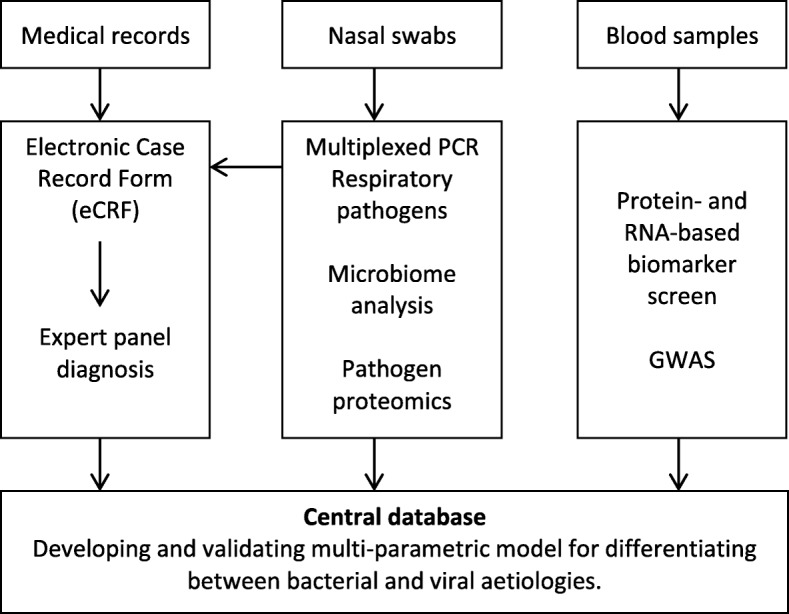
Development of the multi-parametric model

All clinical data (eCRFs) and research data (host biomarkers, host microbiome, pathogen identity and resistance) is coded and uploaded to a central, secured database (HoPOIT database) to which access is granted only to research partners (Fig. 3). The centralized clinical, microbiological, molecular and biochemical data is used to develop a multi-parametric model and diagnostic tools for differentiating between bacterial and viral infections.

### Aetiology determination

Currently, no single reference standard exists for differentiating between bacterial and viral aetiology in an acute infectious disease. Therefore, we follow the Standards for Reporting of Diagnostic Accuracy (STARD) recommendation [39], and create a highly rigorous reference standard based on expert panel adjudication (Fig. 2). The eCRF of each patient is available to a panel of three independent physicians that are affiliated to the country of recruitment. Based on the information included in the eCRF, each member of the panel assigns one of the following diagnostic labels to each one of the patients: (i) bacterial infection; (ii) viral infection; (iii) mixed infection (i.e., bacterial and viral co-infection); (iv) non-infectious disease; or (v) undetermined. Of note, each expert is blinded to the research results and to the labels of his peers on the expert panel. For this purpose, mixed infection (bacterial and viral co-infection) is considered as bacterial infections, as they often elicit similar patient management. A final diagnosis is determined based on consensus agreement between all three experts (Fig. 2). The final diagnosis is used for the purpose of calculating the accuracy of the newly developed diagnostic tools. Cases with only majority agreement (2 out of 3 experts) are analysed as a separate sub-group. The remaining cases are labelled as undetermined. Specifically, this label is assigned in cases where each of the experts assigns a different diagnosis or in cases where 2 or more of the experts assign an “undetermined” diagnosis. These patients are used for data analysis and are expected to comprise roughly 10% of all patients.

### Study stages

The study is conducted in two consecutive stages. Stage A is used to collect molecular, biochemical, microbiological and clinical data on a group of approximately 900 patients. Collected data is used to develop unique diagnostic signatures able to distinguish between bacterial and viral aetiologies, a mass spectrometry based rapid detection technique for identification of microbial pathogens and antimicrobial resistances and a personalized treatment algorithm for tailored antimicrobial therapy that integrates clinical, molecular and biochemical data. In stage B, a second, independent cohort of approximately 300 patients is recruited for the purpose of testing and validating the performances of the multi-parametric model.

### Outcomes

#### Primary endpoint

Sensitivity and specificity for a multi-parametric diagnostic model, incorporating different pathogen- and host-related factors, in differentiating between bacterial and viral aetiology in patients with LRTI and/or sepsis.

#### Secondary endpoints

1. Sensitivity and specificity for host-related individual blood biomarkers, in differentiating between bacterial and viral aetiology from other aetiologist in patients with LRTI and/or sepsis;
2. Sensitivity and specificity for host-related sets of blood biomarkers, in differentiating Gram positive or Gram negative or atypical aetiology from other disease aetiologist in patients with LRTI and/or sepsis;
3. Monitoring the temporal dynamics of host-related blood biomarkers levels during the course of disease in patients with LRTI and/or sepsis;
4. A list of significant bacterial microbiome components that are associated with poor or favourable clinical outcome in patients with LRTI and/or sepsis;
5. Sensitivity and specificity for liquid chromatography-mass spectrometry (LC-MS/MS) and lipid-based Protein Immobilization (LPI) proteomics-based rapid detection technique in identifying pathogens in clinical samples of patients with LRTI and/or sepsis;
6. A web-based application that recommends physicians with a preferred antimicrobial treatment based on patients clinical, molecular and biochemical data;
7. To define genetic mechanisms underlying the different host response between patients with viral versus bacterial infection.

### Sample size calculation

The following power analysis aims to achieve the primary study objective of developing a new diagnostic model for classifying patients with viral and bacterial aetiologist and treatment algorithms to guide antimicrobial prescribing.

#### Paediatric population analysis

First, we estimate the sample size required to reject the null hypothesis that the sensitivity over the entire population, P, is lower than P0 = 70% (H0: P ≤ P0, H1: P > P0) with significance level of 1% (α = 0.01), power of 80% (=1-β) for a true sensitivity P1 of 90% (P1 - P0 ≥ 0.2). For sufficiently large sample sizes (n > ~ 30) the sample distribution of the sensitivity, is approximately normally distributed, ($\widehat{p}$ ~ N{P, P(1-P)/n}). Thus, the number of bacterial patients that are required is:

$$ \mathrm{n}=\frac{{\left({\mathrm{Z}}_{1-\upalpha}\sqrt{{\mathrm{P}}_0\left(1-{\mathrm{P}}_0\right)}+{\mathrm{Z}}_{1-\upbeta}\sqrt{{\mathrm{P}}_1\left(1-{\mathrm{P}}_1\right)}\right)}^2}{{\left({\mathrm{P}}_1-{\mathrm{P}}_0\right)}^2}\le 76 $$

We estimate that the ratio between bacterial and viral paediatric patients is 1:3 and that expert consensus is achieved in 65% of the cases. Thus, the number of patients with an acute infection required in order to reach 76 bacterial patients is 468 = (76 / 25% / 65%). Using similar considerations for computing specificity the sample size for viral patients is also 76. Of note, we anticipate that the above mentioned 468 patients will include 229 (= 468 × 75% × 65%) viral patients, and thus there is no need for additional patients. To assess the host-response based diagnostics accuracy in differentiating patients with an acute infection (both viral and bacterial) and non-infectious patients, assuming P0 = 70% (H0: P ≤ P0, H1: P > P0) with significance level of 1% (α = 0.01), power of 80% (=1-β) for a true sensitivity P1 of 90% (P1 - P0 ≥ 0.2) requires 76 non-infectious patients. Overall, the paediatric cohort should include at least 468 patients with a suspected infectious disease and 76 patients with non-infectious.

#### Adult population analysis

Using the same model as for the paediatric populations (assuming α = 0.01, 1-β = 0.2, P1 = 90%, P0 = 70% and a consensus rate of 65%) and assuming a viral to bacterial rate of 3:7 yields 390 patients with an infectious disease. Additionally, 76 non-infectious patients will be enrolled (based on similar calculations as for the paediatric population).

### Methods for computing the accuracy of a multi-parametric model

The multi-parametric model integrates various host-related and pathogen-related parameters into a single score that determines the likelihood of bacterial and viral aetiology (Fig. 3). The score is computed using a multinomial logistic regression formula that is developed during Stage A of the study. Using predetermined cut-offs, each of the patients is classified as bacterial, viral, or inconclusive (i.e., a marginal immune response that is neither clearly bacterial nor viral). The model diagnosis will be compared with the consensus diagnosis in order to determine its accuracy. The diagnostic accuracy is quantified by computing the following measures: sensitivity, specificity, positive predictive value, negative predictive value, total accuracy, positive likelihood ratio, negative likelihood ratio and diagnostic odds ratio. The area under the receiver operation curve is computed to perform cut-off independent comparisons of different diagnostic methods.

## Discussion and expected results

The inappropriate use of antibiotics has severe global health and economic consequences, including the emergence of antibiotic-resistant bacteria [33]. A major driver of antibiotic misuse is the inability to accurately distinguish between bacterial and viral infections based on currently available diagnostic solutions [33]. Many different biomarkers have been investigated in previous studies, none of them being accurate enough to establish or rule out bacterial infections [40]. The TAILORED-Treatment study is a novel collaboration between (university) hospitals, academic institutions and (diagnostic) industry, aimed to collect and analyse ‘multi-omics’ data from patients suffering from LRTI and/or sepsis. Univariate and multivariate analysis of the data will generate new biomarkers to distinguish between bacterial, viral, and non-infectious disease. The multi-omics approach to generate infectious disease algorithms utilised by this study is currently unique within this area of medicine and provides a template for follow-up combined infectious disease data studies in the future.

Some challenges remain for this type of study. First of all, as described previously, in a subset of patients, sampling of blood and nasal swabs at two sampling points (at presentation and after 3–5 days) should allow to measure data on the temporal dynamics of the host-pathogen interaction. However, if patients are not admitted to the hospital or are already discharged after 1 or 2 days, it is hard to collect a second sample. However, consecutive samples are not necessarily needed in order to achieve the primary objective. A second challenge is how to best interpret and efficiently act on the wealth of data available. Therefore, dedicated bioinformatics professionals are part of the TAILORED-Treatment consortium, for building the unique (HoPOIT) database and to develop the treatment algorithms. Finally, the study is a non-interventional study, and therefore, the diagnostic prediction of the model is not used for any clinical, diagnostic or prognostic decision making. After completing the study, utility studies will be needed to establish the clinical impact of any TAILORED-Treatment algorithms developed.

In summary, the aim of this prospective international (TAILORED-Treatment) study is to develop algorithms to differentiate between bacterial and viral infections in children and adults with LRTI and/or sepsis. The study has the potential to improve patient care, reduce unnecessary antibiotic prescribing (thereby reducing the spread of antibiotic resistances) and will contribute positively to institutional, regional and national healthcare economics.

## Additional files

Additional file 1: Questionnaire follow-up. (DOCX 33 kb)

## Abbreviations

AE: Adverse events
eCRF: Electronic case record form
ED: Emergency Department
EMC: Erasmus University Medical Centre Rotterdam
HAD: Hadassah University Hospital
LRTI: Lower respiratory tract infection
PCR: Polymerase chain reaction
SIRS: Systemic inflammatory response syndrome
UMCU: University Medical Centre Utrecht
