# Supplementary material for: Observational multi-centre, prospective study to characterize novel pathogen-and host-related factors in hospitalized patients with lower respiratory tract infections and/or sepsis - the “TAILORED-Treatment” study
Source: BMC Infect Dis. 2018 Aug 7;18:377. doi: 10.1186/s12879-018-3300-9 (PMC6081806; doi:10.1186/s12879-018-3300-9)
Supplement: Supplementary file 1 — Questionnaire follow-up. (DOCX 33 kb) [file 12879_2018_3300_MOESM1_ESM.docx]

**Questionnaire follow-up**

1) What is diagnosed in the hospital? (initial diagnosis)

1. Lower respiratory tract infection, bronchitis, bronchiolitis or pneumonia
2. Ear infections or colds
3. Infection, otherwise .........
4. The following non-infectious diagnosis: ...................
5. Do not know
6. None

2) Has the patient been hospitalized?

1. No
2. Yes, but not in the Intensive Care
3. Yes, on the Intensive Care
4. Patient is deceased

3) Are there still health problems?

1. YES / NO
2. If YES:
3. Patient is still in hospital
4. Patient is at home, but still has the following symptoms:
   1. Cough: YES / NO
   2. Fever: YES / NO
5. Other: .....................................

4) Are there after the initial diagnosis, other diagnoses? This includes serious incidents or events, such as pneumothorax or a severe allergic reaction.

5) What medication has been used since the start of the study?

1. Patient has antibiotic (AB) used?
   1. NO
   2. Yes:
   3. AB Name: ...................
   4. Patient still uses AB: YES / NO

Patient has antiviral medicines

1. NO
2. YES, namely: ............................
3. Otherwise, name medications: ...........................
4. Do not know
